# Paracoccidioidomycosis Diagnosed in Europe—A Systematic Literature Review

**DOI:** 10.3390/jof7020157

**Published:** 2021-02-23

**Authors:** Gernot Wagner, Deddo Moertl, Anna Glechner, Verena Mayr, Irma Klerings, Casey Zachariah, Miriam Van den Nest, Gerald Gartlehner, Birgit Willinger

**Affiliations:** 1 Department for Evidence-Based Medicine and Evaluation, Danube University Krems, Dr.-Karl-Dorrek-Strasse 30, 3500 Krems, Austria; anna.glechner@donau-uni.ac.at (A.G.); verena.mayr@donau-uni.ac.at (V.M.); irma.klerings@donau-uni.ac.at (I.K.); CaseyZachariah@hotmail.com (C.Z.); gerald.gartlehner@donau-uni.ac.at (G.G.); 2 Clinical Department of Internal Medicine III, University Hospital St. Poelten, Karl Landsteiner University of Health Sciences, Dunant-Platz 1, 3100 St. Poelten, Austria; deddo.moertl@stpoelten.lknoe.at; 3 Department for Infection Control and Hospital Epidemiology, Medical University of Vienna, Waehringer Guertel 18-20, 1090 Vienna, Austria; miriam.vandennest@meduniwien.ac.at; 4 Division of Clinical Microbiology, Department of Laboratory Medicine, Medical University of Vienna, Waehringer Guertel 18-20, 1090 Vienna, Austria; birgit.willinger@meduniwien.ac.at; 5 RTI International, 3040 East Cornwallis Road, P.O. Box 12194, Research Triangle Park, NC 27709-2194, USA

**Keywords:** paracoccidioidomycosis, *Paracoccidioides* spp., endemic systemic mycosis

## Abstract

Paracoccidioidomycosis is a systemic mycosis that is endemic in geographical regions of Central and South America. Cases that occur in nonendemic regions of the world are imported through migration and travel. Due to the limited number of cases in Europe, most physicians are not familiar with paracoccidioidomycosis and its close clinical and histopathological resemblance to other infectious and noninfectious disease. To increase awareness of this insidious mycosis, we conducted a systematic review to summarize the evidence on cases diagnosed and reported in Europe. We searched PubMed and Embase to identify cases of paracoccidioidomycosis diagnosed in European countries. In addition, we used Scopus for citation tracking and manually screened bibliographies of relevant articles. We conducted dual abstract and full-text screening of references yielded by our searches. To identify publications published prior to 1985, we used the previously published review by Ajello et al. Overall, we identified 83 cases of paracoccidioidomycosis diagnosed in 11 European countries, published in 68 articles. Age of patients ranged from 24 to 77 years; the majority were male. Time from leaving the endemic region and first occurrence of symptoms considerably varied. Our review illustrates the challenges of considering systemic mycosis in the differential diagnosis of people returning or immigrating to Europe from endemic areas. Travel history is important for diagnostic-workup, though it might be difficult to obtain due to possible long latency period of the disease.

## 1. Introduction

Paracoccidioidomycosis, also known as South American blastomycosis, is a systemic fungal infection [1] caused by the thermally dimorphic fungi of the species *Paracoccidioides brasiliensis* and the related species *P. americana, P. restrepiensis, P. venezuelensis,* and *P. lutzii* [2,3]. These fungi are endemic to certain geographic regions of Central and South America [4]. Most of the cases of paracoccidioidomycosis are reported in Brazil, followed by Colombia, Venezuela, Ecuador, and Argentina [5]. Based on estimates from epidemiological data, the number of cases of paracoccidioidomycosis in Brazil ranges from 3360 to 5600 per year [5]. The incidence of cases considerably varies among regions with low, moderate or high endemicity [5]. According to estimates, in regions with a stable endemic situation, the annual incidence of paracoccidioidomycosis ranges from 1 to 4 cases per 100,000 inhabitants [5].

People living in rural areas and working in agriculture are particularly at risk for this mycosis [1]. The risk of infection is higher for men than women [6]. The chronic form (adult type) accounts for the majority of cases [4]. This form of paracoccidioidomycosis is progressive over months or years and can be unifocal, if only one site is affected, or multifocal, in case of dissemination [7]. The organ most frequently affected is the lung [7]. Skin, oral mucosa, pharynx, larynx, lymph nodes, adrenal glands, central nervous system, bones, or joints may also be affected [8]. Symptoms of the disease can be systemic (e.g., weight loss, general weakness) or related to specific organ affection (e.g., cough, shortness of breath) [8]. In particular, pulmonary affection, lymphadenopathy, and B symptoms often lead to clinical signs similar to tuberculosis [8,9].

Paracoccidioidomycosis differential diagnosis is particularly challenging, because clinical signs and symptoms, as well as histopathological findings, resemble numerous other infections (e.g., tuberculosis) and noninfectious diseases (e.g., sarcoidosis) [8]. In addition, a long latency period [7] between exposure and manifestation of symptoms, as well as limited clinical experience, make adequate diagnosis difficult. In nonendemic areas, the history of travel and residency in endemic regions is a key to consider paracoccidioidomycosis for differential diagnosis.

Most physicians in nonendemic areas are unfamiliar with the clinical picture of endemic systemic mycoses because they are rarely presented to them. This in turn increases the risk that patients with paracoccidioidomycosis end up with misdiagnosis or remain undiagnosed. Subsequently, this results in no or inappropriate therapy. Therefore, it is important to provide information about the disease presentations in nonendemic regions.

A previously published review by Ajello et al. 1985 [10] comprehensively summarized internationally published cases of paracoccidioidomycosis from Africa, Asia, the Middle East, North America, and Europe [10]. However, this review is now 35 years old and needs to be updated.

The purpose of this systematic review is to summarize the evidence of paracoccidioidomycosis imported to nonendemic European countries. Thereby, we aim to increase awareness for this fungal infection and provide important information regarding its challenging diagnosis.

## 2. Materials and Methods

For reporting of this systematic review, we followed the Preferred Reporting Items for Systematic Reviews and Meta-Analyses statement (PRISMA) [11].

### 2.1. Information Sources and Literature Search

An experienced information specialist searched PubMed and Embase (Embase.com ((accessed on 16 December 2020))) from inception to June 15 and 16, 2020 to identify relevant publications. We used a combination of subject headings and title and abstract free-text terms. We restricted our search to adults and humans. We have provided the detailed search strategy in Appendix A (Table A1 and Table A2). In addition to database searches, we used Scopus (Elsevier) on 16 June 2020 to perform forward and backward citation tracking of included publications and reviews. We also manually screened reference lists of these records, in case the reference lists available via Scopus were incomplete. To identify publications published prior to 1985, we used the previous review published by Ajello et al. [10]. We used references found by our search to identify relevant publications published in 1985 or later.

### 2.2. Eligibility Criteria and Study Selection

Our population of interest was adults of any age and origin diagnosed with paracoccidioidomycosis (South American blastomycosis) in geographic Europe. We considered any case description of an acute or chronic form of paracoccidioidomycosis as eligible for this review if authors provided sufficient clinical information on number of cases, country of exposure, and diagnosis. Publications were included regardless of language and type of publication. We included case series and case reports, observational studies, reviews providing information mentioned above and published as abstracts, full-articles, letters, and editorials. Table 1 provides a summary of eligibility criteria.

After a pilot round, two reviewers independently screened each title and abstract. Eligible publications subsequently underwent independent dual full-text assessment. We solved disagreements by consensus or involvement of a senior reviewer. Throughout the whole study selection process, we used the web-based software Covidence [12]. We organized search and screening results in an EndNote^®^ X9 bibliographic database (Clarivate, PA, USA).

### 2.3. Data Collection Process and Evidence Synthesis

We extracted the following relevant information from each article into pilot-tested evidence tables: author, year, study design, language, country of diagnosis, country of exposure, number of cases, patient characteristics (age, gender, occupation, affected organ(s), systemic antimycotic therapy, and treatment response), and latency period. If the publication language was not English, we asked native speakers to translate or used the online tool DeepL (http://www.deepl.com (accessed on 15 January 2021)) for translations into German. We synthesized data of identified articles narratively.

## 3. Results

### 3.1. Study Selection and Characteristics

**Figure 1:**
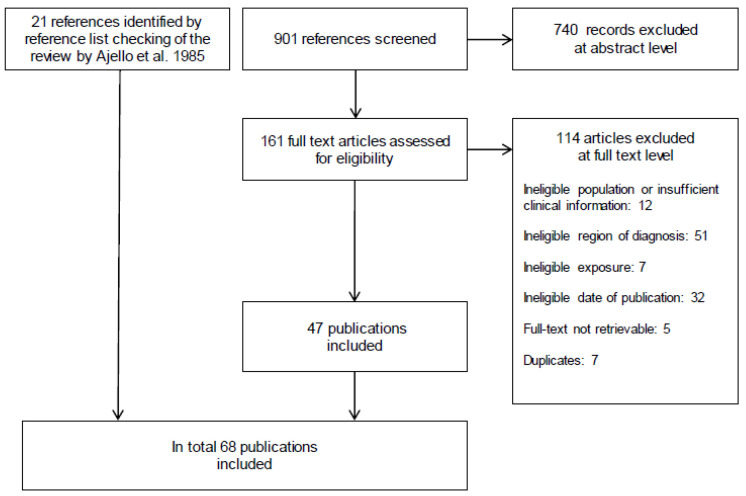
Modified Preferred Reporting Items for Systematic Reviews and Meta-Analyses (PRISMA) flow diagram [11].

Overall, we identified 83 case reports from 11 European countries, published in 68 articles. Figure 1 shows details of the study selection process.

Table 2 summarizes the number of publications and reported cases by country. Spain reported most of the cases, followed by Italy and Germany. The majority of articles were written in English or Spanish. Other publication languages were German, Portuguese, Italian, Norwegian and French.

### 3.2. Clinical Patient Characteristics

The age of the patients ranged from 24 to 77 years. The infection mainly affected men. In most cases, exposure to Paracoccidioides took place in Venezuela, followed by Brazil and Ecuador. The most common occupations were field and construction workers. Latency period, defined as the period from leaving the endemic region until occurrence of first symptoms or medical contact, ranged from six days to 50 years. Table 3 shows patient characteristics, country of exposure, latency period, affected organ(s), systemic antimycotic therapy and response to treatment grouped by countries in which the diagnosis was made.

### 3.3. Differential Diagnosis

Table A3 of Appendix A shows infectious and non-infectious diseases that were considered for differential diagnosis of cases in the included articles.

### 3.4. Diagnostic Work-up

The diagnostic workup varied across publications. Usually, *Paracoccidioides* spp. was identified from clinical specimens through microscopic visualization and/or culture. In addition, some of the authors reported results from serological tests and/or molecular biological techniques such as polymerase chain reaction (PCR). Table A3 provides information on diagnostic workup in individual cases of paracoccidioidomycosis.

In general, direct examination, using 10% potassium hydroxide applied to different samples, is effective and inexpensive. A histologic examination of tissue specimens using silver methenamine or periodic acid-Schiff stain is common and practical when patients present with oral or other skin lesions. In a clinical sample, Paracoccidioides spp. appear as globose yeast cells with multiple buds and a thick refractile wall [81].

## 4. Discussion

Our systematic review summarizes the evidence on published case reports of imported paracoccidioidomycosis diagnosed in Europe. To the best of our knowledge, this is the most recent and comprehensive review of published cases of this systematic mycosis endemic to geographical regions of Central and South America. While narrative reviews on patients with this disease often included a nonsystematic search, we followed a systematic approach with a much broader scope to identify all published cases of paracoccidioidomycosis imported to Europe. In addition, the last systematic assessment of case reports on paracoccidioidomycosis was published in 1985, almost four decades ago [10]. A more recent narrative review focused only on cases diagnosed in Spain [82].

**Figure 2:**
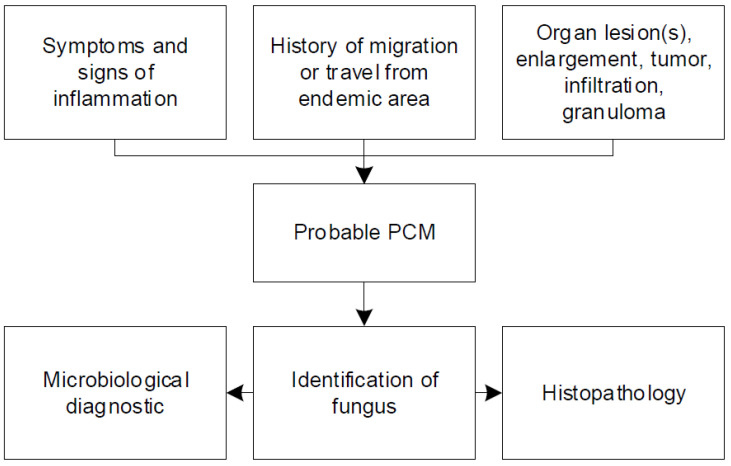
Summary of important aspects for the diagnosis of paracoccidioidomycosis.

Our systematic review of case reports and case series emphasizes the clinical challenges and pitfalls of paracoccidioidomycosis. Most of the physicians in non-endemic regions such as Europe are unfamiliar with systemic mycosis. They struggle with the diagnostic work-up and management due to several reasons. In general, depending on the type, clinical presentation of patients with paracoccidioidomycosis is variable [4]. A major issue is the clinical similarity to several other infectious and non-infectious diseases [81]. Paracoccidioidomycosis is commonly misdiagnosed as tuberculosis [83]. The clinical picture of tuberculosis resembles the chronic progressive form of paracoccidioidomycosis [9]. The differential diagnosis of chronic paracoccidioidomycosis with lung involvement also includes coccidioidomycosis, histoplasmosis, sarcoidosis, pneumoconiosis, interstitial pneumonia, and malignancy [84]. Inappropriate treatment could have harmful consequences for the patient, without any prognostic impact on systemic mycosis. In addition, the latency period from pathogen exposure to development of symptoms is highly variable and might comprise several decades when patients might already have left the endemic region [7]. Therefore, clinicians must inquire about any short- and long-term stay (travel and residency) in endemic areas and even time abroad many years preceding presentation. Figure 2 summarizes important aspects that have to be considered for diagnosis of paracoccidioidomycosis, including signs and symptoms, travel history, and imaging.

If paracoccidioidomycosis is considered for differential diagnosis, clinicians should provide this information to the microbiologist, pathologist and other laboratory personnel to ensure that adequate methods for direct and indirect identification of the pathogen are applied. In addition, laboratory personnel need to apply safety precautions when collected specimens are handled.

The strengths of our work are the systematic literature search and screening. However, this systematic review has several limitations. First, we have not included cases that may have been diagnosed but never published. Second, because translation methods varied, we might have missed relevant information in the articles. A native speaker translated Spanish texts into German but online electronic translation tools provided translations for all other languages (11 publications) except texts published in English and German. Third, our findings rely on not uniformly structured case reports and cases series that are considered as low-level evidence. Finally, although we conducted comprehensive additional literature searches, we might have missed studies not cited in previous reviews and not indexed in electronic databases due to very early publication dates or non-indexed journals.

## 5. Conclusions

In conclusion, this review highlights the importance of considering systemic mycosis in the differential diagnosis of people with symptoms of tuberculosis who have either returned to Europe from endemic areas or were natives of endemic countries who immigrated to Europe. In light of systemic mycosis’s potentially long latency period, extensive evaluation of travel history is an essential key for a quick and correct diagnosis of systematic endemic mycosis such as paracoccidioidomycosis.

## Figures and Tables

**Table 1:** Eligibility criteria.

	Inclusion	Exclusion
Population	Adults of any age and origin Diagnosed with acute or chronic form of paracoccidioidomycosis (South American blastomycosis)	Children and adolescents Any other infections
	Sufficient clinical information on number of cases, country of exposure and diagnosis	Insufficient clinical information
Region	Diagnosis was made in geographic Europe	Diagnosis was made outside geographic Europe
Study design	Case reports and case series Observational studies Reviews	-
Publication type	Any (e.g., abstract, full article, letters, and editorials)	-
Language	Any	-

**Table 2:** Number of identified publications and reported cases of paracoccidioidomycosis by country of diagnosis.

Country of Diagnosis	No. of Publications	No. of Reported Cases
Austria	2	2
Bulgaria	1	1
France	5	5
Germany	8	9
Great Britain	5	6
Italy	15	15
Netherlands	1	1
Norway	1	1
Portugal	7	7
Spain	21	35 ^1^
Switzerland	2	1
Total	68	83

^1^ Based on number of cases reported by Vivancos et al. 1969 [13] (*n* = 1), Pereiro Miguens et al. 1974 [14] (*n* = 2), Simon Merchán, et al. 1970 [15] (*n* = 1), Pereiro Miguens et al. 1987 [16] (*n* = 1), Miguélez et al. 1995 [17] (*n* = 2), Molina-Morant et al. 2018 [18] (*n* = 25), Agirre et al. 2019 [19] (*n* = 2) and Chamorro-Tojeiro et al. 2020 [20] (*n* = 1). Abbreviation: No., number; *n*, number of patients.

**Table 3:** Imported cases of paracoccidioidomycosis from Central and South America diagnosed in Europe.

Author, Year	Study Design Language	No. of Cases	Age Years, Gender	Suspected Country of Exposure	Latency Period ^1^	Occupation	Affected Organ(s)	Systemic Antimycotic Therapy	Treatment Response
AUSTRIA									
Wagner et al. 2016 [21]	Case report English	1	62, M	Peru	6 years	Construction worker	Adrenal glands, brain, lung, lymph node	Amphotericin B, itraconazole, posaconazole	Clinical improvement during hospital stay
Mayr et al. 2004 [22]	Case report English	1	43, F	Brazil, Venezuela or Mexico	4 years	Government employee	Lung, lymph node	Amphotericin B, voriconazole	Clinical improvement
BULGARIA									
Balabanov et al. 1964 [23]	Case report * French	1	67, M	Brazil	30 years	Worked in the jungle	Lung, oral mucosa	Sulfonamide, trimethoprim	Complete remission
GERMANY									
Kayser et al. 2019 [24]	Case report English	1	57, F	Venezuela	1 year	NR	Lung	Amphotericin B, itraconazole	Clinical improvement during hospital stay, remained under control
Slevogt et al. 2004 [25]	Case report English	1	31, F	Brazil	10 years	NR	Lymph nodes	Itraconazole	Complete remission
Horré et al. 2002 [26]	Case report English	1	61, M	Brazil	10 years	Legionnaire	Lung, oral mucosa, skin	Itraconazole	Complete remission
Köhler et al. 1988 [27]	Case report German	1	49, M	Brazil	15 years	Gold mine worker	Brain, lung, oral and laryngeal mucosa	Amphotericin B, ketoconazole, itraconazole	Clinical improvement during hospital stay, remained under control
Neveling 1988 [28]	Case series German	3	38, F	Brazil	1 month	Administrative employee	Lung	None	Clinical improvement, remained under control
			64, M	Brazil	1 month	Gardener	Lung	NR	NR
			45, M	Brazil	1 month	Librarian	Lung	NR	NR
Braeuninger et al. 1985 [29], Hastra et al. 1985 [30]	Case report German	1	32, M	Peru	6 years	NR	Lung, lymph nodes, oral mucosa, skin	Ketoconazole	Clinical improvement
Altmeyer 1976 [31]	Case report * German	1	69, M	Paraguay	22 years	Farm worker	Lung, lymph nodes, oral mucosa, skin	Imidazole	Patient deceased
PORTUGAL									
Ferreira et al. 2017 [32]	Case report English	1	46, M	Brazil	1 month	NR	Lung, lymph nodes, oral mucosa	Itraconazole	Clinical improvement
Coelho et al. 2013 [33]	Case report (Abstract only) English	1	63, M	Brazil	8 years	Gardener	Oral and pharyngeal mucosa	NR	NR
Alves et al. 2013 [34]	Case report Portuguese	1	43, M	Venezuela	6 years	NR	Lung, oral mucosa, skin	Itraconazole	Complete remission
Armas et al. 2012 [35]	Case report English	1	43, M	Venezuela	NR	Farm worker	Lung, skin	Itraconazole	Clinical improvement
Carvalho et al. 2009 [36]	Case report Portuguese	1	24, M	Brazil	7 years	Construction worker	Lymph nodes, skin	Itraconazole	Clinical improvement
Villar et al. 1963 [37]	Case report **	1	-	Brazil	37 years	-	-	-	-
Oliveira et al. 1960 [38]	Case report ** Portuguese	1	-	Brazil	23 years	-	-	-	-
SPAIN									
Chamorro-Tojeiro et al. 2020 [20]	Case report English	1	42, M	Mexico	6 days	NR	Lung	Itraconazole	Complete remission, remained under control
Agirre et al. 2019 [19]	Case series English	2	29, F	Peru	10 days	NR	Lung, lymph nodes	Itraconazole	Complete remission
			31, M	Peru	10 days	NR	Lung	Itraconazole	Complete remission
Molina-Morant et al. 2018 [18]	Retrospective observational study English	25 ^2^	Median 48 yrs (range 33 to 67), M 16 (64%)	This retrospective study reported 25 cases of paracoccidioidomycosis admitted to Spanish hospitals between 1 January 1997 to 31 December 2014.					
Navascués et al. 2013 [39]	Case report Spanish	1	47, M	Ecuador	11 years	NR	Lung, lymph nodes, skin	Amphotericin B, itraconazole	Complete remission
Buitrago et al. 2011 [40]	Case series English	6	67, M	Ecuador			NR		
			57, M	Venezuela			NR		
			44, M	Paraguay			NR		
			51, M	Paraguay			NR		
			31, M	Ecuador			NR		
			NR, M	NR			NR		
Pujol-Riqué et al. 2011 [41]	Case report Spanish	1	48, M	Brazil	6 years	Worked in the jungle	Lung	Amphotericin B, itraconazole	Complete remission
Ramírez-Olivencia et al. 2010 [42]	Case report English	1	56, M	Venezuela	6 months	NR	Lung	Itraconazole, amphotericin B	Complete remission
Botas-Velasco et al. 2010 [43]	Case report Spanish	1	43, M	Ecuador	NR	NR	Lung, oral and laryngeal mucosa	Itraconazole	Clinical improvement; disappearance of oral and laryngeal lesions
Mayayo et al. 2007 [44]	Case report English	1	27, M	Ecuador	None	Farmer	Lymph nodes, skin	Itraconazole	Complete remission
López Castro et al. 2005 [45]	Case report Spanish	1	63, M	Venezuela	8 months	Shoemaker	Lung, skin	Amphotericin B	Patient deceased
Ginarte et al. 2003 [46]	Case series English	3	72, M	Venezuela	50 years	NR	Skin	Fluconazole	Remained under control
			67, M	Brazil	1 year	NR	Oral mucosa, teeth	Itraconazole, sulfonamides	Complete remission
			65, M	Venezuela	38 years	NR	Oral mucosa	Fluconazole	Remained under control
Garcia Bustínduy et al. 2000 [47]	Case report English	1	59, M	Venezuela	1 year	Taxi driver	Skin	Itraconazole	Complete remission
Del Pozo et al. 1998 [48]	Case report Spanish	1	50, M	Venezuela	13 years	NR	Skin, oral and nasal mucosa	Itraconazole	Complete remission
Garcia et al. 1997 [49]	Case report Spanish	1	72, M	Venezuela	50 years	NR	Lung, oral mucosa, skin	Fluconazole	Complete remission
Pereiro et al. 1996 [50]	Case report English		This case was also described by Ginarte et al. 2003 [46] and is therefore not presented here again.						
Miguélez et al. 1995 [17]	Case report Spanish	2	44, M	Venezuela	2 years	NR	Brain, lung, lymph nodes	Itraconazole	Patient deceased (tuberculosis coinfection)
			53, M	Venezuela	18 months	NR	Lung, lymph nodes, oral mucosa	Itraconazole	Clinical improvement
Pereiro Miguens et al. 1987 [16]	Case report Spanish	1	51, M	Venezuela	23 years	Construction worker	Oral mucosa, skin	Ketoconazole	Clinical improvement
Simon Merchán et al. 1970 [15]	Case report **	1	-	Venezuela	2 years	-	-	-	-
Pereiro Miguens 1974 [14], Pereiro Miguens et al. 1972 [51]	Case report * Spanish	2	44, M	Venezuela	7 years	NR	Lung, oral mucosa	Sulfonamide, trimethoprim	Clinical improvement
			49, M	Venezuela	8 years	Gardener	Oral mucosa	Sulfonamide, trimethoprim	Clinical improvement
Vivancos et al. 1969 [13]	Case report * Spanish	1	44, M	Venezuela	NR	Farmer	Oral mucosa	Sulfamethoxazole/trimethoprim, amphotericin B	Complete remission
GREAT BRITAIN									
De Cordova et al. 2012 [52]	Case report (Abstract only) English	1	52, M	Venezuela	NR	Butcher	Oral mucosa	Itraconazole	Complete remission
Sierra et al. 2011 [53]	Case report (Abstract only) English	1	77, M	Ecuador	NR	NR	Lung, lymph nodes, oral mucosa, skin	Itraconazole	NR
Walker et al. 2008 [54]	Case report English	1	51, M	Venezuela	None	Accountant	Lung, oral mucosa, skin	Itraconazole	Clinical improvement, cutaneous lesions cleared
Bowler et al. 1986 [55]	Case report English	1	57, F	Argentina, Venezuela	17 years	Clerk	Lung	NR	NR
Symmers 1966 [56]	Case report * English	1	42, M	Brazil	NR	Engineer	Skin	NR	NR
		1	46, M	Brazil	None	Businessman	Spleen (autopsy)	No therapy	Patient deceased (acute heart failure)
ITALY									
Borgia et al. 2000 [57]	Case report English	1	61, M	Venezuela	NR	House- painter	Bones, lung	Itraconazole	Clinical improvement
Pecoraro et al. 1998 [58]	Case report Italian	1	60, M	Venezuela	NR	Coffee plantations worker	Bones, lung	Ketoconazole	Clinical improvement
Solaroli et al. 1998 [59]	Case report Italian	1	49, M	Brazil	NR	NR	Brain, lung, skin	Itraconazole	Clinical improvement
Fulciniti et al. 1996 [60]	Case report English	1	60, M	Venezuela	NR	NR	Bones, lung	Itraconazole	Clinical improvement, remained under control
Cuomo et al. 1985 [61]	Case report Italian	1	37, M	Venezuela	2 years	Butcher	Lung, skin	Ketoconazole	Clinical improvement
Benoldi et al. 1985 [62]	Case report * English	1	41, M	Venezuela	Few months	Butcher	Lung, lymph nodes, skin	Ketconazole, sulfamethoxy-pyridazine	Complete remission
Finzi et al. 1980 [63]	Case report **	1	-	Brazil	14 years	-	-	-	-
Velluti et al. 1979 [64]	Case report * Italian	1	52, M	Venezuela	17 years	Fabric retailer	Lung	Amphotericin B, miconazole	Clinical improvement
Lasagni et al. 1979 [65]	Case report ** Italian	1	-	Venezuela	NR	-	-	-	-
Scarpa et al. 1965 [66]	Case report * Italian	1	43, M	Venezuela	5 years	Farmer	Lung, oral mucosa, skin	Amphotericin B, sulfamethoxazole	Patient deceased
Schiraldi et al. 1963 [67]	Case report **	1	-	Venezuela	None	-	-	-	-
Molese et al. 1956 [68]	Case report * Italian	1	47, M	Venezuela	None	Painter	Lung, lymph nodes, oral mucosa	Nystatin	NR
Farris 1955 [69]	Case report **	1	-	Brazil	7 years	-	-	-	-
Bertaccini 1934 [70]	Case report **	1	-	Brazil	None	-	-	-	-
Dalla Favera 1914 [71]	Case report **	1	-	Brazil	None	-	-	-	-
FRANCE									
Heleine et al. 2020 [72]	Case report English	1	48, M	Brazil	NR	Farmer	Lung, lymph nodes, oropharyngeal mucosa, skin	Itraconazole	Clinical improvement
Dang et al. 2017 [73]	Case report English	1	54, M	Columbia, Venezuela	12 years	Journalist	Lymph nodes, oropharyngeal mucosa	Itraconazole	Clinical improvement; almost complete resolution of the tongue lesion and lymhadenopathy
Sambourg et al. 2014 [74]	Case report French	1	43, M	Brazil	NR	NR	Skin	NR	NR
Laccourreye et al. 2010 [75]	Case report English	1	46, M	Venezuela	NR	Engineer	Laryngeal mucosa	Itraconazole	Complete remission
Poisson et al. 2007 [76]	Case report English	1	70, M	Paraguay	6 years	NR	Brain, lung	Fluconazole, itraconazole	Remained clinically stable
NETHERLANDS									
Van Damme et al. 2006 [77]	Case report English	1	60, M	Peru, Ecuador	8 years	Carpenter	Lung, oral mucosa, urinary tract	Itraconazole	Clinical improvement, remained under control
NORWAY									
Maehlen et al. 2001 [78]	Case report Norwegian	1	51, F	Brazil	23 years	NR	Brain	-	Patient deceased
SWITZERLAND									
Stanisic et al. 1979 [79], Wegmann et al. 1959 [80]	Case report * German	1	47, M	Brazil	5 years	Carpenter	Lung, lymph nodes, oral mucosa	Hydroxy-stilbamidine, amphotericin B, sulfonamide	Patient deceased (Cor pulmonale)

**Abbreviations:** M, male; F, female; NR, not reported; yrs, years; ^1^ Latency period from leaving the endemic region until occurrence of first symptoms or medical contact; ^2^ We assume that most of 25 cases diagnosed in Spain between 1997 to 2014 and published by Molina-Morant et al. 2018 [18] are also described in case reports and case series presented in this table; * Included in the review by Ajello et al. 1985 [10]; ** Included in the review by Ajello et al. 1985, full-text not available, data extracted from Ajello et al. 1985 [10].

## Data Availability

No new data were created or analyzed in this study. Data sharing is not applicable to this article.

## List of Abbreviations

PCR	polymerase chain reaction
PCM	paracoccidioidomycosis
PRISMA	Preferred Reporting Items for Systematic Reviews and Meta-Analyses

## Appendix A

**Table A1:** Search Strategy Pubmed 15 June 2020.

Search Number	Query	Results
1	“Paracoccidioidomycosis” [Mesh]	1833
2	Paracoccidioidomycos* [tiab]	1782
3	Paracoccidioides brasiliensis [tiab]	1611
4	paracoccidioidal granuloma [tiab]	9
5	south american blastomycosis [tiab]	272
6	#1 OR #2 OR #3 OR #4 OR #5	2858
7	“Europe” [Mesh]	1,408,827
8	“Emigrants and Immigrants” [Mesh]	12,277
9	“Travel” [Mesh:NoExp]	24,916
10	(Albania* [tiab] OR Andorra* [tiab] OR Armenia* [tiab] OR Austria* [tiab] OR Azerbaijan* [tiab] OR Belarus* [tiab] OR Belgi* [tiab] OR Bosnia* [tiab] OR Herzegov* [tiab] OR Bulgaria* [tiab] OR Croatia* [tiab] OR Cypr* [tiab] OR Czech [tiab] OR Denmark [tiab] OR danish [tiab] OR Estonia* [tiab] OR Finland [tiab] OR finnish [tiab] OR France [tiab] OR french [tiab] OR Georgia* [tiab] OR German* [tiab] OR Greece [tiab] OR greek [tiab] OR Hungar* [tiab] OR Iceland* [tiab] OR Ireland [tiab] OR irish [tiab] OR Italy [tiab] OR italian [tiab] OR Kazak* [tiab] OR Kosov* [tiab] OR Latvia* [tiab] OR Liechtenstein* [tiab] OR Lithuania* [tiab] OR Luxembourg* [tiab] OR Macedonia* [tiab] OR Malta [tiab] OR maltese [tiab] OR Moldov* [tiab] OR Monac* [tiab] OR Montenegr* [tiab] OR Netherlands [tiab] OR dutch [tiab] OR Norway [tiab] OR norwegian [tiab] OR Poland [tiab] OR polish [tiab] OR Portug* [tiab] OR Romania* [tiab] OR Russia* [tiab] OR San Marino [tiab] OR Serbia* [tiab] OR Slovakia* [tiab] OR Slovenia* [tiab] OR Spain [tiab] OR spanish [tiab] OR Sweden [tiab] OR swedish [tiab] OR Switzerland [tiab] OR swiss [tiab] OR Turkey [tiab] OR turkish [tiab] OR Ukrain* [tiab] OR United Kingdom [tiab] OR britain [tiab] OR british [tiab])	1,082,126
11	(Albania* [ad] OR Andorra* [ad] OR Armenia* [ad] OR Austria* [ad] OR Azerbaijan* [ad] OR Belarus* [ad] OR Belgi* [ad] OR Bosnia* [ad] OR Herzegov* [ad] OR Bulgaria* [ad] OR Croatia* [ad] OR Cypr* [ad] OR Czech [ad] OR Denmark [ad] OR danish [ad] OR Estonia* [ad] OR Finland [ad] OR finnish [ad] OR France [ad] OR french [ad] OR Georgia* [ad] OR German* [ad] OR Greece [ad] OR greek [ad] OR Hungar* [ad] OR Iceland* [ad] OR Ireland [ad] OR irish [ad] OR Italy [ad] OR italian [ad] OR Kazak* [ad] OR Kosov* [ad] OR Latvia* [ad] OR Liechtenstein* [ad] OR Lithuania* [ad] OR Luxembourg* [ad] OR Macedonia* [ad] OR Malta [ad] OR maltese [ad] OR Moldov* [ad] OR Monac* [ad] OR Montenegr* [ad] OR Netherlands [ad] OR dutch [ad] OR Norway [ad] OR norwegian [ad] OR Poland [ad] OR polish [ad] OR Portug* [ad] OR Romania* [ad] OR Russia* [ad] OR San Marino [ad] OR Serbia* [ad] OR Slovakia* [ad] OR Slovenia* [ad] OR Spain [ad] OR spanish [ad] OR Sweden [ad] OR swedish [ad] OR Switzerland [ad] OR swiss [ad] OR Turkey [ad] OR turkish [ad] OR Ukrain* [ad] OR United Kingdom [ad] OR britain [ad] OR british [ad])	5,875,438
12	europ* [tiab] OR immigrant* [tiab] OR travel* [tiab]	383,712
13	non endemic [tiab] OR nonendemic [tiab]	4492
14	#13 OR #12 OR #11 OR #10 OR #9 OR #8 OR #7	7,173,281
15	#6 AND #14	204
16	(“Animals” [Mesh] NOT “Humans” [Mesh])	4,707,502
17	#15 NOT #16	175

**Table A2:** Search Strategy Embase 16 June 2020.

No.	Query	Results
#1	‘south american blastomycosis’/exp OR ‘paracoccidioides brasiliensis’/exp	3026
#2	paracoccidioidomycos*:ab,ti OR ‘paracoccidioides brasiliensis’:ab,ti OR ‘paracoccidioidal granuloma’:ab,ti OR ‘south american blastomycosis’:ab,ti	3075
#3	#1 OR #2	3620
#4	‘europe’/exp OR ‘immigrant’/exp OR ‘travel’/exp	1,695,885
#5	albania*:ca,ab,ti OR andorra*:ca,ab,ti OR armenia*:ca,ab,ti OR austria*:ca,ab,ti OR azerbaijan*:ca,ab,ti OR belarus*:ca,ab,ti OR belgi*:ca,ab,ti OR bosnia*:ca,ab,ti OR herzegov*:ca,ab,ti OR bulgaria*:ca,ab,ti OR croatia*:ca,ab,ti OR cypr*:ca,ab,ti OR czech:ca,ab,ti OR denmark:ca,ab,ti OR danish:ca,ab,ti OR estonia*:ca,ab,ti OR finland:ca,ab,ti OR finnish:ca,ab,ti OR france:ca,ab,ti OR french:ca,ab,ti OR georgia*:ca,ab,ti OR german*:ca,ab,ti OR greece:ca,ab,ti OR greek:ca,ab,ti OR hungar*:ca,ab,ti OR iceland*:ca,ab,ti OR ireland:ca,ab,ti OR irish:ca,ab,ti OR italy:ca,ab,ti OR italian:ca,ab,ti OR kazak*:ca,ab,ti OR kosov*:ca,ab,ti OR latvia*:ca,ab,ti OR liechtenstein*:ca,ab,ti OR lithuania*:ca,ab,ti OR luxembourg*:ca,ab,ti OR macedonia*:ca,ab,ti OR malta:ca,ab,ti OR maltese:ca,ab,ti OR moldov*:ca,ab,ti OR monac*:ca,ab,ti OR montenegr*:ca,ab,ti OR netherlands:ca,ab,ti OR dutch:ca,ab,ti OR norway:ca,ab,ti OR norwegian:ca,ab,ti OR poland:ca,ab,ti OR polish:ca,ab,ti OR portug*:ca,ab,ti OR romania*:ca,ab,ti OR russia*:ca,ab,ti OR ‘san marino’:ca,ab,ti OR serbia*:ca,ab,ti OR slovakia*:ca,ab,ti OR slovenia*:ca,ab,ti OR spain:ca,ab,ti OR spanish:ca,ab,ti OR sweden:ca,ab,ti OR swedish:ca,ab,ti OR switzerland:ca,ab,ti OR swiss:ca,ab,ti OR turkey:ca,ab,ti OR turkish:ca,ab,ti OR ukrain*:ca,ab,ti OR ‘united kingdom’:ca,ab,ti OR britain:ca,ab,ti OR british:ca,ab,ti	10,276,132
#6	europ* OR ‘non endemic’ OR nonendemic OR travel*:ab,ti OR imported:ti	2,203,591
#7	immigrant*	34,244
#8	#4 OR #5 OR #6 OR #7	11,651,119
#9	#3 AND #8	457
#10	‘animal’/exp NOT ‘human’/exp	5,449,241
#11	#9 NOT #10	405
#12	‘groups by age’/exp NOT ‘adult’/exp	2,775,185
#13	#11 NOT #12	397
#14	‘case report’/exp OR ‘case study’/exp OR ‘letter’/exp	3,471,553
#15	case:ab,ti OR cases:ab,ti	4,628,697
#16	‘review’/exp OR ‘evidence based medicine’/exp	3,575,830
#17	review:ab,ti OR systematic:ab,ti OR search*:ab,ti OR ‘meta analy*’:ab,ti OR metaanaly*:ab,ti	2,588,995
#18	#14 OR #15 OR #16 OR #17	10,847,515
#19	#13 AND #18	256

**Table A3:** Signs and symptoms, differential diagnosis and diagnostic work-up.

Author, Year	Symptoms and Signs	Differential Diagnosis	Specimen for Histopathology	Histo-Logy ^1^	Micro-Biology ^2^	Sero-Logy	PCR
AUSTRIA							
Wagner et al. 2016 [21]	Chest and abdominal pain, weight loss, night sweats, cough	Tuberculosis	Left adrenal gland biopsy, extirpation of a right cervical lymph node	+	+	NR	+
Mayr et al. 2004 [22]	Cough, lymphadenopathy, weight loss	Tuberculosis, Wegener’s granulomatosis, sarcoidosis, mycosis	Lung biopsy	+	+	+	NR
BULGARIA							
Balabanov et al. 1964 [23]	Ulcerous oral and cutaneous lesions, lymphadenopathy	Tuberculosis	Peribuccal lesion biopsy	+	+	NR	NR
GERMANY							
Kayser et al. 2019 [24]	Cough, dyspnea	Sarcoidosis, histoplasmosis	Lung biopsy	+	+	+	+
Slevogt et al. 2004 [25]	Bilateral cervical and axillary lymphadenopathy, weight loss	Tuberculosis	Cervical lymph node biopsy	+	+	NR	NR
Horré et al. 2002 [26]	Erythematous and swollen lips, mucocutaneous pustules and ulcerations, oral nodules, occasional night sweats	Leishmaniosis, tropical pulmonary mycosis, gammopathy	Oral lesion biopsy	+	+	+	+
Köhler et al. 1988 [27]	Cheilitis, erosive stomatitis, loss of teeth, dysphagia, aphonia, cough, night sweats, weight loss	Tropical disease	NR	NR	+	+	NR
Neveling 1988 [28]	Flue like symptoms, dry cough	Coccidiosis, histoplasmosis, North American blastomycosis	NR	NR	NR	+	NR
	NR	NR	NR	NR	NR	+	
	NR	NR	NR	NR	NR	+	
Braeuninger et al. 1985 [29], Hastra et al. 1985 [30]	Flue like symptoms, cervical lymphadenopathy, skin lesions, cough, dyspnea, pain in the left leg	Tuberculosis, sarcoidosis	Lymph node biopsy	+	+	+	NR
Altmeyer 1976 [31]	Respiratory insufficiency, cervical lymphadenopathy, painful infiltrations of the soft palate, hypersalivation, ulcerations of the feet, weight loss, dysphagia, dysphonia	Tuberculosis, Wegner’s granulomatosis	Lung and skin lesion biopsy	+	−	NR	NR
PORTUGAL							
Ferreira et al. 2017 [32]	Labial lesion, dry cough, inguinal and axillary lymphadenopathy, weight loss	Cryptococcosis	Lip lesion and lung biopsy, inguinal lymph node resection	+	−	NR	+
Coelho et al. 2013 [33]	Odynophagia, dysphagia, irregular and ulcerated oral mucosa	NR	Oropharyngeal mucosa biopsy	+	NR	NR	NR
Alves et al. 2013 [34]	Skin lesion, oral mucosal ulcerations	Coccidioidomycosis, cutaneous tuberculosis	Skin lesion and oral mucosa biopsy	+	+	NR	NR
Armas et al. 2012 [35]	Ulcerated skin and nasal mucosa lesion	NR	Skin lesion biopsy	+	+	NR	NR
Carvalho et al. 2009 [36]	Fever, epigastric pain, anorexia, fatigue, lymphadenopathy, skin lesions		Skin biopsy and lymph node	+	NR	NR	NR
Villar et al. 1963 [37]	Full-text not available						
Oliveira et al. 1960 [38]	Full-text not available						
SPAIN							
Chamorro-Tojeiro et al. 2020 [20]	Fever, arthralgia, myalgia, dyspnea, dry cough, sweating, general cutaneous rash	Bacterial respiratory infection	NR	NR	−	+	NR
Agirre et al. 2019 [19]	Fever, productive cough, exertional dyspnea	Bacterial respiratory infection	NR	NR	−	+	NR
	Fever, myalgia, asthenia	NR	NR	NR	−	+	NR
Molina-Morant et al. 2018 [18]	NR	NR	NR	NR	NR	NR	NR
Navascués et al. 2013 [39]	Productive cough, weight loss, asthenia, lymphadenopathy, skin lesions	NR	Lung biopsy	+	−	+	+
Buitrago et al. 2011 [40] ^3^	Fever, asthenia, ulcerated pustular skin lesions, extremities	NR	Skin biopsy	+	NR	+	+
	Productive cough	NR	NR	+	NR	+	+
	NR	NR	Cerebral biopsy	+	NR	+	+
	NR	NR	NR	+	NR	+	+
	NR	NR	Lung biopsy	NR	+	NR	+
	NR	NR	Oral mucosa biopsy	+	NR	+	+
Pujol-Riqué et al. 2011 [41]	Productive cough, hemoptysis, night sweats, skin lesions	Sarcoidosis	Lung and skin biopsy	−	+	NR	NR
Ramírez-Olivencia et al. 2010 [42]	Fever, dyspnea, productive cough, hemoptysis, night sweats, loss of appetite, weight loss	NR	Lung biopsy	NR	−	+	+
Botas-Velasco et al. 2010 [43]	Cough, fever, weight loss, retromolar mass	Sarcoidosis	Retromolar mass and laryngeal biopsy	+	+	+	NR
Mayayo et al. 2007 [44]	Skin lesions	Blastomycosis	Skin lesion biopsy	+	NR	NR	NR
López Castro et al. 2005 [45]	Dyspnea, dry cough, fever, weight loss, skin lesions	Sarcoidosis	Lung and skin biopsy	+	NR	−	NR
Ginarte et al. 2003 [46]	Ulcerative lesions from upper left jaw to labial mucosa and nasal grave	Squamous cell carcinoma	Lesion biopsy	+	+	+	NR
	Ulcerative lesions left cheek mucosa, periodontitis with loss of several teeth	Tuberculosis, squamous cell carcinoma	Lesion biopsy	+	+	NR	NR
	Mass and ulcerative lesions in cheek mucosa	Squamous cell carcinoma	Lesion biopsy	+	+	NR	NR
Garcia Bustínduy et al. 2000 [47]	Ulcerative skin lesion	NR	Skin lesion biopsy	+	+	+	NR
Del Pozo et al. 1998 [48]	Lesions upper labial mucosa and nasal fossa	NR	Lesion biopsy	+	+	NR	NR
Garcia et al. 1997 [49]	Lesions of labial and palatal mucosa	NR	Lesion biopsy	+	+	+	NR
Pereiro et al. 1996 [50]	Tumoral mass of the upper jaw, ulcerated lesion in the upper left jaw, extended to the lip mucosa and the nasal grave	Epidermoid carcinoma	Lesion biopsy	+	+	+	NR
Miguélez et al. 1995 [17]	Fever, weight loss, dyspnea, ulcerated mass right tonsil, lymphadenopathy	Pulmonary fibrosis	Ulcerated mass biopsy	+	+	NR	NR
	Palatal mass, cervical lymphadenopathy	NR	Palatal mass biopsy	+	+	NR	NR
Pereiro Miguens et al. 1987 [16]	Oral mucosal lesions, gingivitis	Tuberculosis	Mucosa biopsy	+	+	+	NR
Simon Merchán et al. 1970 [15]	Full-text not available						
Pereiro Miguens et al. 1974 [14], Pereiro Miguens et al. 1972 [51]	Epididymitis, gingivitis, oral ulcerative lesion	Tuberculosis	Epididymis and oral lesion biopsy	+	+	+	NR
	Asthenia, ulcerative oral lesions, labial edema	NR	Oral lesion biopsy	+	+	NR	NR
Vivancos et al. 1969 [13]	Oral mucosal lesions	Pseudoneoplasia	Oral lesion biopsy	+	+	NR	NR
GREAT BRITAIN							
De Cordova et al. 2012 [52]	Submandibular mass, oral ulcerative lesions	NR	Oral lesion and submandibular mass biopsy	+	+	NR	NR
Sierra et al. 2011 [53]	Dyspnea, lip lesion, ulcer on tonsil and uvula	Malignancy, sarcoidosis, squamous cell carcinoma	Lip lesion excision, ulcer biopsy	+	NR	+	NR
Walker et al. 2008 [54]	Cough, dyspnea, plantar pruritus, painful skin lesions on his legs, face and feet, hepatomegaly, weight loss	NR	Skin biopsy	+	+	+	NR
Bowler et al. 1986 [55]	Cough, dyspnea, and wheeze on exertion	Lymphangitis carcinomatosa	Lung biopsy	+	NR	+	NR
Symmers 1966 [56]	Asymptomatic	NR	Spleen (autopsy)	+	NR	NR	NR
	Skin ulceration	NR	Skin lesion excision	+	NR	NR	NR
ITALY							
Borgia et al. 2000 [57]	Fever, pain, and inflammation of left knee	Malignancy	Left femur biopsy	+	+	NR	NR
Pecoraro et al. 1998 [58]	Weight loss, night sweat, pain left knee	NR	Left femur biopsy	+	NR	NR	NR
Solaroli et al. 1998 [59]	Skin lesion, asthenia, fever, loss of vision	NR	Skin lesion excision	+	+	NR	NR
Fulciniti et al. 1996 [60]	Weight loss, night, sweats, pain in left knee	Metastatic lung cancer	Left femur biopsy	+	+	NR	NR
Cuomo et al. 1985 [61]	Productive cough, weight loss, asthenia, skin lesions	Tuberculosis, lupus vulgaris	Lung and skin lesion biopsy	+	+	+	NR
Benoldi et al. 1985 [62]	Ulcerative skin lesions, cough, fatigue, malaise, weight loss	Tuberculosis, lupus vulgaris	Skin lesion biopsy	+	+	+	NR
Finzi et al. 1980 [63]	Full-text not available						
Velluti et al. 1979 [64]	Cough, dyspnea	Bronchitis, tuberculosis	Lung biopsy	+	+	+	NR
Lasagni et al. 1979 [65]	Full-text not available						
Scarpa et al. 1965 [66]	Cough, asthenia, weight loss, night sweats, lymphadenitis, ulcerative oral lesions	Tuberculosis	Lymph node and lung biopsy	+	+	NR	NR
Schiraldi et al. 1963 [67]	Full-text not available						
Molese et al. 1956 [68]	Oral mucosa lesions, cervical lymphadenopathy, fever, cough	Tuberculosis, leishmaniosis, pneumoconiosis, lues, malignancy	Oral mucosa and tonsillar biopsy	+	NR	NR	NR
Farris 1955 [69]	Full-text not available						
Bertaccini 1934 [70]	Full-text not available						
Dalla Favera 1914 [71]	Full-text not available						
FRANCE							
Heleine et al. 2020 [72]	Skin lesions, ulcero-nodular lesions lips and mouth, cough, fever, inguinal lymphadenopathy, asthenia, weight loss	HIV, tuberculosis	Skin biopsy	−	+	NR	NR
Dang et al. 2017 [73]	Nodular slightly painful, nonulcerated lesion of the tongue, cervical lymphadenopathy	NR	Lingual lesion biopsy	+	NR	NR	+
Sambourg et al. 2014 [74]	Partially ulcerous and crusted erythematous lesion left auricle extending to the pre-auricular region	Leishmaniosis	Skin lesion biopsy	+	+	−	+
Laccourreye et al. 2010 [75]	Dysphonia, laryngitis	Chronic laryngitis	Laryngeal biopsy, removed mucosa	+	+	NR	NR
Poisson et al. 2007 [76]	Seizures	Brain tumor	Single cerebral lesion surgically excised	+	+	+	NR
NETHERLANDS							
Van Damme et al. 2006 [77]	Dyspnea, cough, wheezing, weight loss, tiredness, fever, night sweats, periodontitis, oral ulceration, macrohematuria	Sarcoidosis, bronchiolitis obliterans organizing pneumonia, oral carcinoma	Lung and oral mucosa biopsy	+	+	+	NR
NORWAY							
Maehlen et al. 2001 [78]	Dizziness, nausea, headache, hearing loss, hemiplegia	Cerebral tuberculosis	Brain biopsy	+	+	NR	NR
SWITZERLAND							
Stanisic et al. 1979 [79], Wegmann et al. 1959 [80]	Submandibular and cervical lymphadenopathy, oral ulceration	Tuberculosis, Morbus Wegener, lues, bartonellosis, Morbus Hodgkin, neoplasma, blastomycosis, sporotrichosis, cryptococcosis	Oral mucosa and cervical lymph node biopsy	+	+	NR	NR

**Abbreviations:** NR, not reported or not performed; +, positive for *Paracoccidioides* spp.; −, negative for *Paracoccidioides* spp.; ^1^ Fungal structures were identified in at least one of the biopsy/excision specimen; ^2^ Microbiology includes microscopy and/or culture; ^3^ Signs and symptoms obtained for case 1 and case 2 obtained from Buitrago et al. 2009 [85].
